# Immune cell infiltration and the genes associated with ligamentum flavum hypertrophy: Identification and validation

**DOI:** 10.3389/fcell.2022.914781

**Published:** 2022-08-10

**Authors:** Yang Duan, Songjia Ni, Kai Zhao, Jing Qian, Xinyue Hu

**Affiliations:** ^1^ Department of Spine Surgery, Zhujiang Hospital, Southern Medical University, Guangzhou, Guangdong, China; ^2^ Department of Orthopaedic Trauma, Zhujiang Hospital, Southern Medical University, Guangzhou, Guangdong, China; ^3^ Neurosurgery Department, The Second Affiliated Hospital of Kunming Medical University, Kunming, China; ^4^ Department of Clinical Laboratory, Kunming First People’s Hospital, Kunming Medical University, Kunming, China

**Keywords:** ligamentum flavum hypertrophy, differentially expressed genes, immune cell infiltration, bioinformatics, gene expression omnibus database

## Abstract

Ligamentum flavum hypertrophy (LFH) is a common cause of spinal stenosis. The aim of the current study was to identify the differentially expressed genes (DEGs) in LFH and the molecular mechanisms underlying the development of and immune responses to LFH. The gene expression omnibus (GEO) database was used to obtain the GSE113212 dataset, and the DEGs were derived from microarray data. To identify critical genes and signaling pathways, gene ontology enrichment, Kyoto Encyclopedia of Genes and Genomes (KEGG) enrichment, and protein-protein interaction (PPI) network analyses were performed, followed by immune cell infiltration and Friends analyses using the retrieved datasets. The results were validated using quantitative real-time PCR. The 1530 DEGs identified comprised 971 upregulated and 559 downregulated genes. KEGG analysis revealed that DEGs were mostly enriched in the PI3K-Akt signaling pathway, while PPI network analysis identified tumor necrosis factor, interleukin (IL)-6, IL-10, epidermal growth factor receptor, and leptin as important nodes, which was validated by qPCR and IHC in human LFH tissues *in vitro*. A significant positive correlation was found between key LFH immune-related DEGs and several immune cell types, including T and B cells. The findings of the present study might lead to novel therapeutic targets and clinical approaches, as they provide insights into the molecular mechanisms of LFH.

## Introduction

Ligamentum flavum hypertrophy (LFH) is a major cause of spinal stenosis, a disorder that causes sensory and motor dysfunction in the upper and lower limbs due to compression of the spinal cord and spinal nerve roots or cauda equine syndrome (Pan et al., 2021). The common symptoms of limb numbness in LFH are caused by the abnormal compression of spinal nerves. Amyloid deposition causes the ligamentum flavum to thicken, resulting in spinal canal compression and constriction, and it is likely to occur in 45%–96% of elderly patients undergoing spinal stenosis surgery (Ruberg et al., 2019). Spinal stenosis is the most frequent spinal ailment in elderly adults. LFH is a primary factor in lumbar spinal stenosis (LSS), (Zheng et al., 2020) a type of degenerative lesion that develops with age. Changes in the intervertebral discs, ligamentum flavum, and small joints cause spinal stenosis with age, resulting in leg and back pain, impaired walking, and other symptoms commonly observed in aging individuals or patients with degenerative spine (Wang et al., 2020). LFH is therefore a complex clinical issue, and the identification of potential molecules and underlying mechanisms of LFH is critical to elucidating potential therapeutic targets and improving its prognosis.

A recent study investigated Use of extracellular vesicles made from umbilical cord mesenchymal stromal cells to treat ligamentum flavum hypertrophy (Ma et al., 2023). The macrophage migration inhibitory factor can be linked to LFH in type 2 diabetes (Cisneros et al., 2016). Patients with lumbar spinal stenosis have hypertrophic ligamentum flavum cells, and SIRT6 promotes telomerase activity to prevent DNA damage and senescence (Chen et al., 2021). Fibrillogenic activity is mediated by TGFβ-1 through the Smad proteins, and we have observed that Smad2 knockdown influences the TGFβ-1signaling pathway by lowering TGFβ-1 levels (Wang et al., 2021). The role of the p38 mitogen-activated protein kinase (MAPK) signaling pathway in developing human lumbar LFH is mediated by transforming growth factor 1 (TGF1/connective tissue growth factor) (Lu et al., 2019). Inflammatory cytokines and mechanical stress-induced fibrosis can be reduced by downregulating the CRLF1 gene. Furthermore, in LF mice, standing on both feet increased the expression of HLF and CRLF1 (Zheng et al., 2020). Protease inhibitors called tissue inhibitors of matrix metalloproteinases (TIMPs) prevent the breakdown of the extracellular matrix. On magnetic resonance scans from one study, the ligamentum flavum’s thickness was assessed at the articular eminence level. In addition, the ligamentum flavum tissue’s expression of markers for cell growth and death was investigated. The findings revealed that higher levels of protease inhibitors were linked to ligamentum flavum hypertrophy in lumbar spinal stenosis (Park et al., 2005).

A recent study examined 30 patients with LSS, in which a group of 15 cases of LFH was compared to a control group using immunohistochemical detection of transforming growth factor (TGF) (Amudong et al., 2017). While the LFH group showed disordered fiber alignment, the control group showed regular fiber alignment, suggesting LFH was closely linked to TGF expression. In addition, LFH-induced cytokine receptor-like factor 1 (CRLF1) overexpression has been demonstrated in several studies using a combination of transcriptomics and proteomics of the human ligamentum flavum (LF), followed by immunohistochemistry and real-time polymerase chain reaction (Zheng et al., 2020). High-throughput and bioinformatics technologies may be able to shed light on the underlying pathophysiology of LFH and expose the important molecular pathways involved in the disease.

The aim of the present study was used to compare microarray data between groups with hypertrophied (elderly) and normal (young) LF using the GSE113212 dataset, identify the biological processes involved using gene ontology (GO) enrichment and gene set enrichment analysis (GSEA), and analyze immune cell infiltration in both groups using the CIBERSORT algorithm. These analyses aimed to disclose the biological mechanisms and novel biomarkers of LFH, emphasizing the role of immune cells in the etiology of LFH. Our findings contribute to the elucidation of LFH molecular pathways and establishment of its genetic network, providing a basis for potential therapeutic targets.

## Materials and methods

### Data collection and assessment of differentially expressed genes

Data on gene expression profiles were obtained from the Agilent-039494 SurePrint G3 Human GE v2 8×60K Microarray 039381 (Agilent Technologies, Inc., Santa Clara, CA, United States) and all samples in GSE113212 (Kaufman et al., 1971) of yellow ligaments dataset, retrieved from the gene expression omnibus (GEO) database (Barrett et al., 2007) using the *GEOquery* package (Davis and Meltzer, 2007) in R (https://www.r-project.org). Gene expression profiles were classified into four gene expression subgroups in normal LF (young group) and four gene expression subgroups in hypertrophied LF (elderly group).

Gene expression values were standardized using the “normalize between arrays” function of the *limma* package in R to assess differences in gene expression levels between the two groups (young vs. elderly). The *limma* package (Ritchie et al., 2015) was also used to examine group variances based on data grouping information. Genes with log fold-change (FC) > 1 and *p*-value < 0.05 were classified as upregulated genes, while genes with logFC < −1 and *p*-value < 0.05 were classified as downregulated genes.

Additionally, the differentially expressed genes (DEGs) linked to LFH immunity were obtained by intersecting the DEGs and immune-related genes from the Immunology Database and Analysis Portal (ImmPort; https://www.immport.org/home) (Bhattacharya et al., 2014), which contains 1793 immune-related genes, including the above mentioned DEGs.

### Functional enrichment analysis of differentially expressed genes

Functional annotation of the DEGs into the biological process (BP), molecular function (MF), and cellular component (CC) categories of GO is a common method for performing large-scale functional enrichment analyses of genes. The Kyoto Encyclopedia of Genes and Genomes (KEGG) is a well-known database that stores data on genomes, biological pathways, diseases, and medications. The *clusterprofiler* package (Yu et al., 2012) in R was used to perform the GO term and KEGG pathway enrichment analyses of the DEGs related to LFH, considering *p* < 0.05 as significant.

### Gene set enrichment analysis

To determine the contribution of a given gene set to the phenotype, GSEA is usually performed, as it allows examining the distribution trend of the genes according to their phenotypic associations (Subramanian et al., 2005). We retrieved the “c2.kegg.v7.4.symbols” gene set from the MSigDB database and subjected it to GSEA using the *clusterprofiler* package in R with false discovery rate (FDR) < 0.25 and |normalized enrichment score (NES)| > 1 as the thresholds for associations (Liberzon et al., 2015).

### Gene set variation analysis

Gene set variation analysis (GSVA) is an enrichment method used to analyze pathway activity variations in a simple population in an unsupervised manner. By transforming gene expression matrices across samples into pathway activation score matrices to examine whether distinct metabolic pathways are enriched across samples, GSVA quantifies gene enrichment and allows for subsequent statistical analysis. Variance analysis of GSVA results was carried out using the *limma* package in R to uncover gene sets that were significantly different. We obtained the “c2.kegg.v7.4.symbols” gene set from the MSigDB database for conducting GSVA of the dataset (Hänzelmann et al., 2013). The R package *limma* was then used for comparing the elderly and young groups, considering adjusted *p* < 0.01 as significant.

### Protein–protein interaction

Protein–protein interaction (PPI) networks are formed of individual proteins involved in various processes, such as biological signaling pathways, regulation of gene expression, energy metabolism, and cell cycle regulation, through mutual interactions. Systematic analysis of these interactions is important to understand how proteins work in biological systems, the response mechanisms of biological signaling and metabolism in specific physiological states, and the functional connections between proteins. The Search Tool for the Retrieval of Interacting Genes/Proteins (STRING) database (Szklarczyk et al., 2019) was used to develop a PPI network for the LFH immunity-related DEGs, which was visualized using Cytoscape software (https://cytoscape.org). The PPI network was then evaluated using the clusters devised by Molecular Complex Detection (MCODE) in Cytoscape to find significant modules and genes. DEGs with score > 10 were considered as key DEGs associated with LFH immunity.

### Immune cell infiltration analysis

The CIBERSORT algorithm was used to analyze the immune-cell infiltration in elderly hypertrophied and young normal LF tissues (Chen et al., 2018). The *ggplot2* R package was used to display the distribution of immune cells as box plots, and the Pearson’s correlation coefficient was used to evaluate the relationships between the various immune cells. The R package *corrplot*was used to generate a heatmap.

Single sample GSEA (ssGSEA) allows quantifying the infiltrated immune cells and the activity of specific immune factors. The absolute enrichment of a given gene set is indicated by computing the enrichment fraction of each gene set in each sample. We obtained the genes related to immune infiltrating cells from the literature (Zhang et al., 2020) and performed ssGSEA using the *GSVA* R package. The association between the main immune-related LFH DEGs and immune infiltrating cells was evaluated by Spearman’s correlation analysis. Heatmaps were visualized using the R package *ggplot2*.

### Friends analysis

The Friends analysis approach assesses the functional correlation between different genes in a pathway, suggesting that a gene is more likely to be expressed if it interacts with other genes in the same pathway, and it is widely used to identify critical genes. To identify critical DEGs in LFH, the R package *GOSemSim* (Yu, 2020) was used to calculate the functional correlations between the major DEGs linked with LFH immunity.

### Human ligamentum flavum sample collection

This study used ligamentum flavum tissue, which had the informed consent of the patient’s family and was approved by the Ethics Committee of Zhujiang Hospital of Southern Medical University (Guangzhou, China). Ethical approval number is 2021-KY-122-01. Ligamentum flavum samples were isolated from the anatomical region (L4/5) of LDD (Lumbar disc degeneration) patients. Ligamentum flavum samples acquired from LDD patients with ligamentum flavum thickening (≤4 mm) were considered to be the non-ligamentum flavum hypertrophy (non-HLF) group, whereas the pathological ligamentum flavum samples harvested from LDD patients with ligamentum flavum thickening (> 4 mm) were assigned to the HLF group. The patients’ clinical characteristics are shown in Table1. And Raw data can be found in Supplementary Tables S1, S2.

**TABLE 1:** Clinical data of patients in two groups.

Description/Group	Sequencing of genome-wide SNP and DNA methylation			Verification of genome-wide SNP and DNA methylation		
	Non-HLF (*n* = 5)	HLF (*n* = 5)	*p*-value	Non-HLF (*n* = 10)	HLF (*n* = 10)	*p*-value
Age (year)	58 ± 5	75 ± 5	0.058	52 ± 5	69 ± 3	0.02
Gender (male/female)	2/3	1/4	>0.05	5/5	2/8	>0.05
Lumbar level	L4/5	L4/5	>0.05	L4/5	L4/5	>0.05
LF thickness	0.28 ± 0.01	0.52 ± 0.03	6.12E-05	0.31 ± 0.01	0.52 ± 0.02	6.29E-09

### Quantitative reverse transcription-polymerase chain reaction

The DNAMAN software was used for designing the primers for quantitative reverse transcription-polymerase chain reaction (qRT-PCR) of the selected genes by Sangon Biotech (Shanghai, China). Primer sequences for each gene are listed in Table 2. We used a total of 12 tissues, including 6 cases of ligamentum flavum hypertrophy and 6 cases of non-ligamentum flavum hypertrophy.LFH and normal LF tissues were shredded with scissors and treated with the TriZol reagent (#15596-026; Invitrogen, Waltham, MA, United States) to extract total RNA after ultrasonic fragmentation. Complementary DNA was obtained using the PrimeScript™ RT reagent Kit with gDNA Eraser (#RR047A; TaKaRa Bio Inc., Shiga, Japan). The SYBR Green qPCR Mix (#D7260; Beyotime Biotech Inc., Shanghai, China) was used for qRT-PCR in the 7500 Real-Time PCR System (Thermo Fisher Scientific, Waltham, MA, United States), which was operated in 40 cycles. The *GAPDH* gene was used as the internal reference, and the relative expression of each gene to that of *GAPDH* was calculated using the 2^−**∆∆**CT^ method.

**TABLE 2:** Sequences of the qRT-PCR primers used for each gene.

Gene	Forward primer sequence	Reverse primer sequence
*EGFR*	AGG​CAC​GAG​TAA​CAA​GCT​CAC	ATG​AGG​ACA​TAA​CCA​GCC​ACC
*LEP*	TGC​CTT​CCA​GAA​ACG​TGA​TCC	CTC​TGT​GGA​GTA​GCC​TGA​AGC
*TNF-α*	CTC​AGC​AAG​GAC​AGC​AGA​GGA​C	TGG​AGC​CGT​GGG​TCA​GTA​TGT
*IL-6*	CCT​GAA​CCT​TCC​AAA​GAT​GGC	TTC​ACC​AGG​CAA​GTC​TCC​TCA
*IL-10*	TCA​AGG​CGC​ATG​TGA​ACT​CC	GAT​GTC​AAA​CTC​ACT​CAT​GG
*GAPDH*	AAG​TAT​GAC​AAC​AGC​CTC​AAG	TCC​ACG​ATA​CCA​AAG​TTG​TC

### Immunohistochemical assessments

LF tissues were fixed in 10% neutral formalin and embedded in paraffin. Serial sections of tissues (4 μm thick) were stained with H&E, Masson’s Trichrome and immunohistochemically stained according to a three-step immunoperoxidase method. All antibodies used in immunohistochemical staining include Monoclonal rabbit anti-human Anti-EGFR antibody [EP38Y] (ab52894); Anti-IL-6 antibody [EPR23819-103] (ab290735); Anti-IL-10 antibody [EPR1114] (ab215975); Anti-Leptin antibody (ab117751); Anti-TNF Receptor II antibody [EPR1653] (ab109322) (Abcam, Cambridge, MA, United States) for 1:100 following the standard staining protocols. Nonspecific isotype IgG was used as a negative control. The absence of staining due to technical failure was excluded by including appropriate positive control tissues (breast carcinoma) in each staining run.

The densities and intensities of cells with positive staining for *EGFR, IL-6, IL-10, LEP*, and *TNF-α* were determined using manual counting by two independent trained investigators (S.N. and J.Q.) who were blinded to the clinical data. The densities and intensities are given as cells per square millimeter by semi-quantitative score method.

### Statistical analyses

Data analyses and statistical tests were performed in R (https://www.r-project.org/, version 4.0.2). For comparison of continuous variables, significance differences of normally distributed variables were calculated by the independent Student’s *t*-test, and differences between non-normally distributed variables were evaluated by the Mann-Whitney U-test (i.e., Wilcoxon rank-sum test). Receiver operating characteristic (ROC) curves were drawn using the *ROC R* package, (Sing et al., 2005) and the area under the curve (AUC) was determined to assess the potential for discriminating disease and control groups. All statistical *p* values were two-tailed, and *p* < 0.05 was considered significant.

## Results

### Data normalization and distribution

**FIGURE 1:**
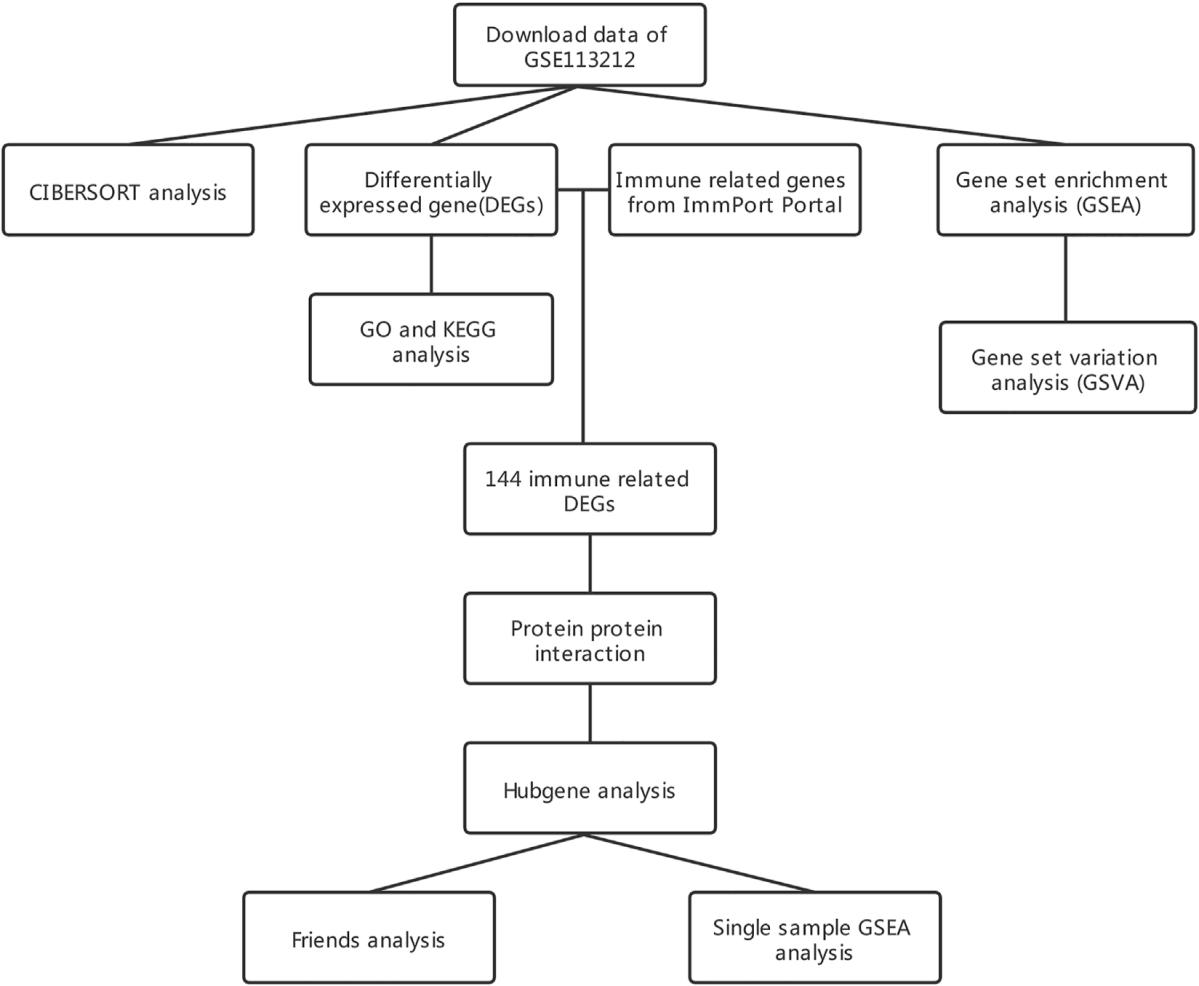
Flowchart of the study design.

**FIGURE 2:**
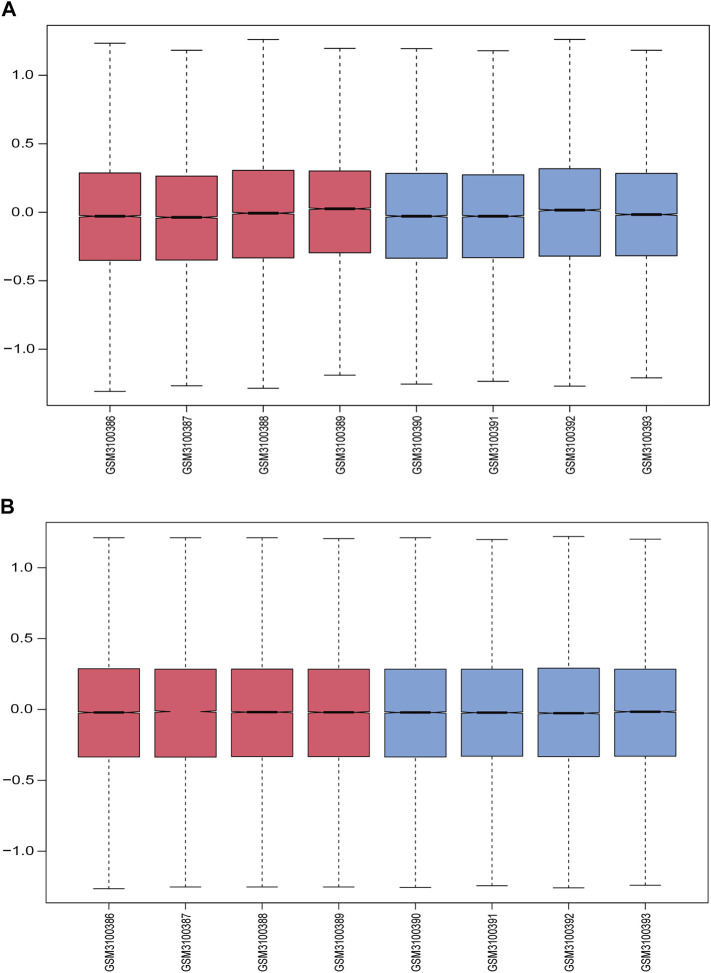
Data normalization. **(A)** Gene expression levels in each sample before normalization are shown in box plots; red indicates the elderly LFH group, and blue indicates the young normal LF group. **(B)** Gene expression levels in each sample after normalization.

Figure 1 displays the study design and data processing as a flowchart. To investigate the variability of gene expression in the two study groups (elderly LFH tissue and young normal LF tissue), the gene expression levels were first standardized. A comparable distribution was then observed in the gene expression values of both groups (Figures 2A,B).

### Immune cell infiltration analysis

**FIGURE 3:**
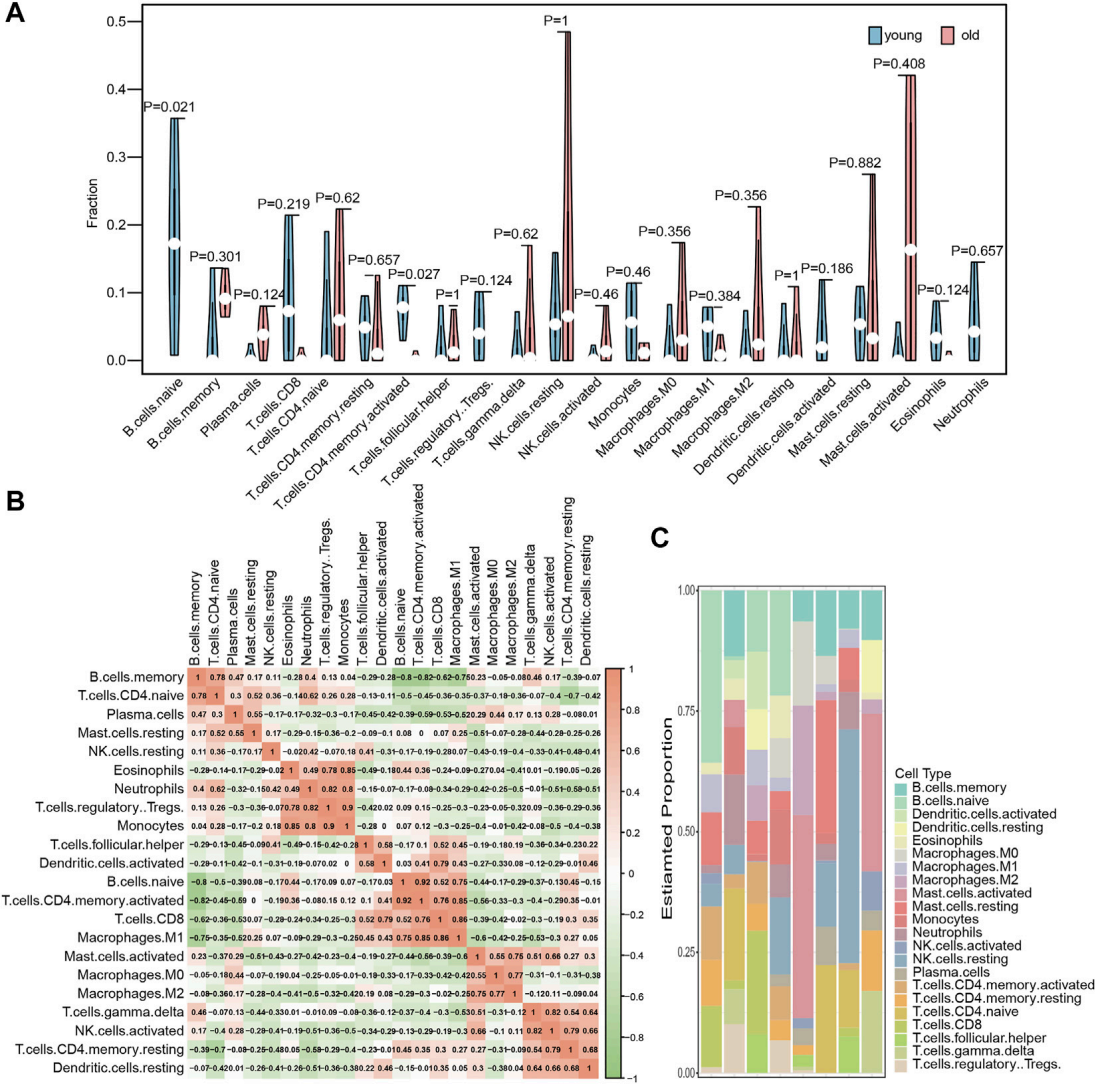
Immune cell infiltration analysis. **(A)** Differences in the abundance of 22 immune cell types enriched in the GSE113212 dataset; green indicates the young normal LF group, red indicates the elderly LFH group; the horizontal axis indicates the 22 immune cell types, and the vertical axis the abundance of immune cell infiltration. **(B)** Correlation analysis between the various immune cell types in the GSE113212 dataset; color intensity represents the strength of correlation−the more red or green the color, the stronger the correlation between two immune cell types. **(C)** Stacked bar graphs of the 22 immune cell types in different samples of the GSE113212 dataset; different color bars represent different immune cell types.

We used the CIBERSORT algorithm in the expression profile data to calculate the degree of infiltration of 22 immune cell types in the elderly LFH and young normal LF groups. The proportion of two immune cell types, namely, memory activated CD4 T cells and B cells, in the GSE113212 dataset showed significant differences between the LFH and normal LF groups (Wilcoxon test, *p* < 0.05) (Figure 3A). The infiltration of memory activated CD4 T cells and naïve B cells were significantly lower in the LFH group than in the normal LF group, suggesting that these two types of immune cells play a key role in the process of LFH. The proportion of the remaining 20 immune cell types did not differ significantly between the two groups. The memory activated CD4 T cells showed significant positive (*p* < 0.05) correlations with naïve B cells (Figure 3B), M1 macrophages, and CD8 T cells. The relative proportions of immune cell types in the different samples are displayed in Figure 3C.

### Differentially expressed genes

**FIGURE 4:**
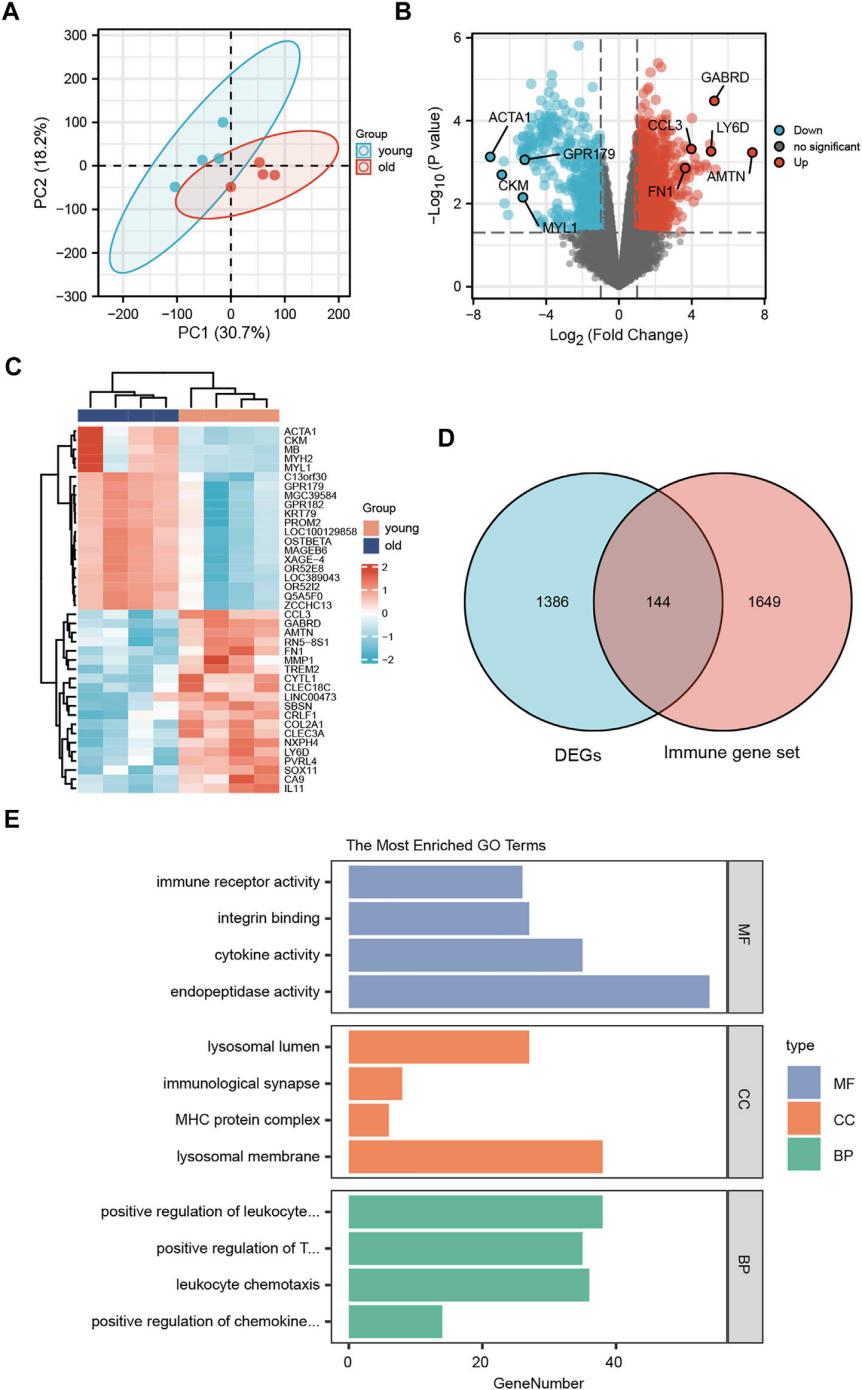
Differentially expressed genes. **(A)** Principal component analysis of the GSE113212 dataset. **(B)** Horizontal coordinates are log2FC, vertical coordinates are −log10 (adjusted p); red nodes indicate upregulated genes, blue nodes indicate downregulated genes, gray nodes indicate genes that are not significantly differentially expressed. **(C)** Horizontal coordinates are patient ids, vertical coordinates are their DEGs; red represents genes with high expression, blue represents genes with low expression, pink annotation bar indicates young normal LF group, blue annotation bar indicates elderly LFH group. **(D)** Blue circles indicate DEGs in GSE113212, pink circles indicate immune-related gene sets; the intersection shows LFH immune-related DEGs. **(E)** GO functional enrichment analysis; vertical coordinates are the various GO terms enriched for DEGs, and horizontal coordinates are the number of DEGs enriched in each GO term.

To assess the reproducibility of the data in GSE113212, a principal component analysis was performed (Figure 4A). To analyze the gene expression values of the group with LFH (elderly group) relative to that of the control group (young group with normal LF tissue) and obtain the DEGs, we used the *limma* R package. The volcano plot obtained for 1530 identified DEGs in the GSE113212 dataset revealed that 971 DEGs were upregulated while 559 DEGs were downregulated (Figure 4B). The heatmap was drawn by clustering the DEGs (Figure 4C) revealed these could clearly distinguish between LFH and normal tissues.

The list of 1793 immune-related genes downloaded from the ImmPort database was overlapped with the identified DEGs to obtain the immune-related DEGs of LFH, which are presented in the Wayne diagram shown in Figure 4D.

### Functional enrichment analysis of differentially expressed genes

To analyze the relationship between the DEGs associated with LFH and the GO categories BP, CC, and MF, we first performed functional enrichment analysis of these DEGs (Figure 4E; Table 3). Leukocyte activation, positive regulation of T cell activation, leukocyte chemotaxis, and positive regulation of chemokine production in BP, lysosomal membrane, MHC protein complex, immunological synapse, lysosomal lumen, and immune receptor activity in CC, and integrin binding, endopeptidase activity, and cytokine activity in MF were enriched.

**TABLE 3:** GO enrichment analysis of DEGs.

Term	ID	Description	p.adjust
BP	GO:0030198	Extracellular matrix organization	8.94E-21
BP	GO:0043062	Extracellular structure organization	8.94E-21
BP	GO:0051216	Cartilage development	2.71E-08
BP	GO:0001503	Ossification	6.06E-08
BP	GO:0061448	Connective tissue development	9.46E-08
BP	GO:0030199	Collagen fibril organization	2.45E-07
BP	GO:1903039	Positive regulation of leukocyte cell-cell adhesion	1.48E-05
BP	GO:0050870	Positive regulation of T cell activation	3.01E-05
BP	GO:0030595	Leukocyte chemotaxis	5.40E-05
BP	GO:0032722	Positive regulation of chemokine production	5.74E-05
CC	GO:0062023	Collagen-containing extracellular matrix	1.71E-27
CC	GO:0005788	Endoplasmic reticulum lumen	5.57E-19
CC	GO:0005581	Collagen trimer	2.35E-11
CC	GO:0043202	Lysosomal lumen	1.73E-09
CC	GO:0005775	Vacuolar lumen	3.57E-08
CC	GO:0005583	Fibrillar collagen trimer	1.78E-07
CC	GO:0098643	Banded collagen fibril	1.78E-07
CC	GO:0042613	MHC class II protein complex	0.003610522
CC	GO:0005765	Lysosomal membrane	0.014640391
CC	GO:0001772	Immunological synapse	0.018111925
MF	GO:0005201	Extracellular matrix structural constituent	2.66E-16
MF	GO:0019838	Growth factor binding	3.09E-08
MF	GO:0030020	Extracellular matrix structural constituent conferring tensile strength	4.24E-08
MF	GO:0140375	Immune receptor activity	6.03E-05
MF	GO:0005178	Integrin binding	6.03E-05
MF	GO:0005125	Cytokine activity	0.000273783
MF	GO:0004175	Endopeptidase activity	0.000273783
MF	GO:0004896	Cytokine receptor activity	0.000273783
MF	GO:0005518	Collagen binding	0.000613795
MF	GO:0005539	Glycosaminoglycan binding	0.000914208

**FIGURE 5:**
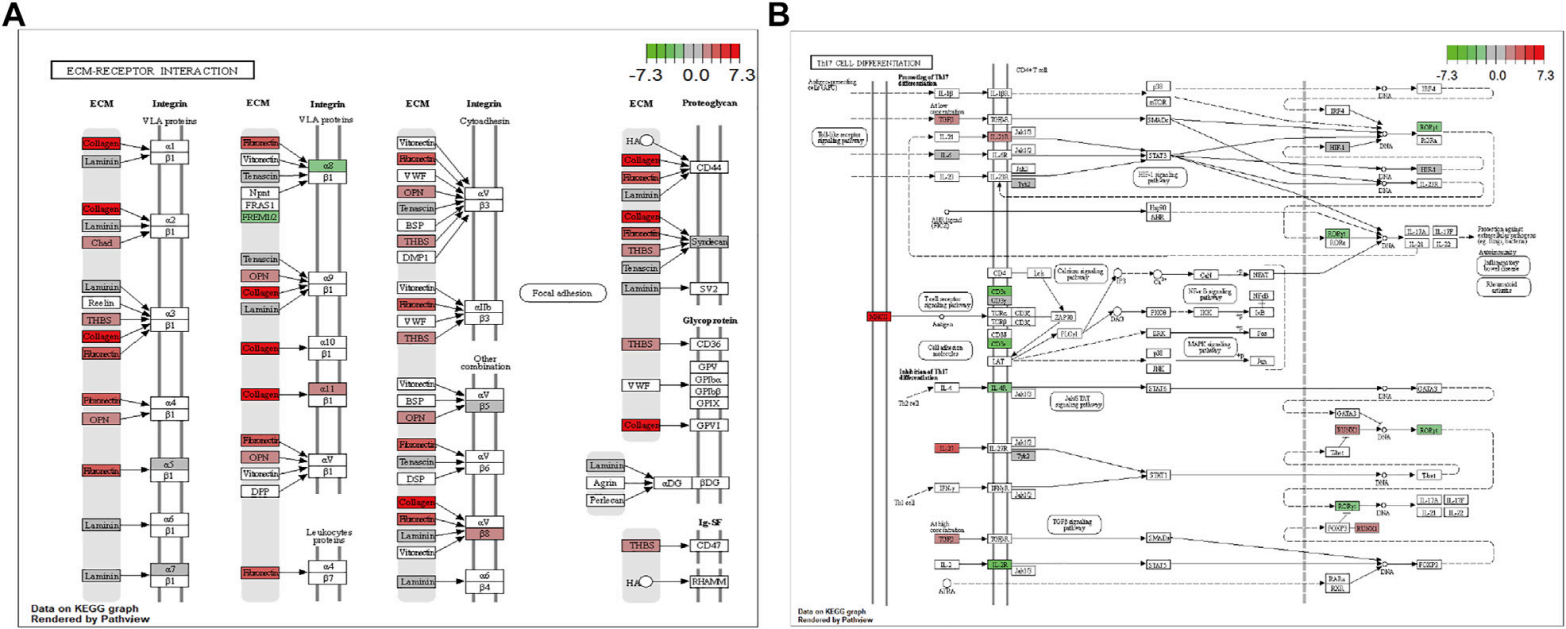
Visualization of the enriched KEGG pathways. **(A)** ECM-receptor interaction. **(B)** Th17 cell differentiation pathway. Each node indicates a gene that plays an important role in the pathway, and the color of the node is related to the log2FC value; green indicates downregulated genes, and red indicates upregulated genes.

In addition, pathway enrichment analysis indicated DEGs were enriched in ECM-receptor interaction, protein digestion and absorption, cytokine-cytokine receptor interaction, PI3K-Akt signaling pathway, and Th17 cell differentiation, among other biological pathways. The most significantly enriched pathways were ECM-receptor interaction (hsa04512, Figure 5A) and the immune-related pathway Th17 cell differentiation (hsa04659, Figure 5B).

### Gene set enrichment analysis and gene set variation analysis

**FIGURE 6:**
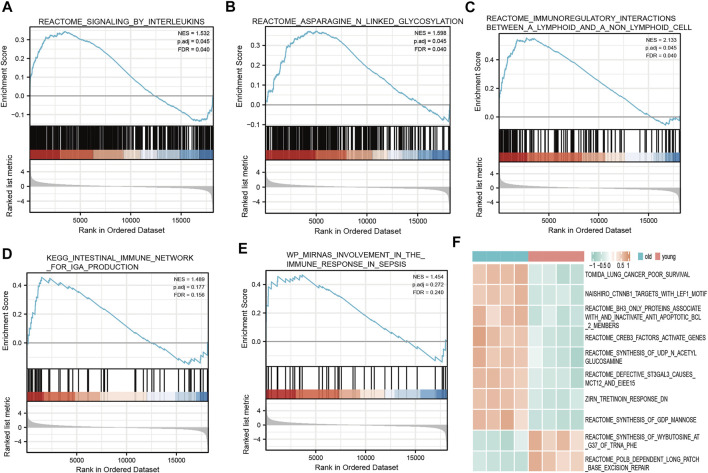
GSEA and GSVA. GSEA revealed the following Reactome pathways were enriched in the elderly LFH group: **(A)** Signaling by interleukins; **(B)** asparagine linked glycosylation; **(C)** immunoregulatory interactions between a lymphoid and a non-lymphoid cell. The enriched KEGG pathways were: **(D)** Intestinal immune network for IgA production; and **(E)** miRNA involvement in the immune response in sepsis. **(F)** Results of GSVA. Pink annotation bars indicate the young normal LF group, blue annotation bars indicate the elderly LFH group. The heatmap shows the GSVA scores for each pathway in this sample.

We determined which biological pathways were most affected by differences in gene expression levels in the LFH and normal tissue datasets. In the GSE113212 dataset, the most affected Reactome pathways were signaling by interleukins, asparagine-linked glycosylation, immunoregulatory interactions between a lymphoid and a non-lymphoid cell; the most affected KEGG pathways were intestinal immune network for IgA production and micro RNA involvement in the immune response in sepsis, among other biologically relevant pathways (Figures 6A–E). Thus, in LFH tissue, some immune-related signaling pathways were enriched, suggesting that immune factors may play an important role in the development of this disease.

The GSVA performed to determine the enrichment of biological pathways in the LFH group and normal LF group showed the most affected Reactome pathways, among other biologically relevant pathways, in the disease group: *CREB3* factors activate genes, *BH3* only proteins associate with and inactivate anti-apoptotic *BCL2* members, synthesis of N-acetyl members, synthesis of acetyl glucosamine, and defective *ST3GAL3* causes *MCT12* and *EIEE15* (Figure 6F). In the normal LF group, the Reactome pathway POLB-dependent long patch base excision repair was mainly affected (Figure 6F), among other relevant pathways.

### Protein–protein interaction network

**FIGURE 7:**
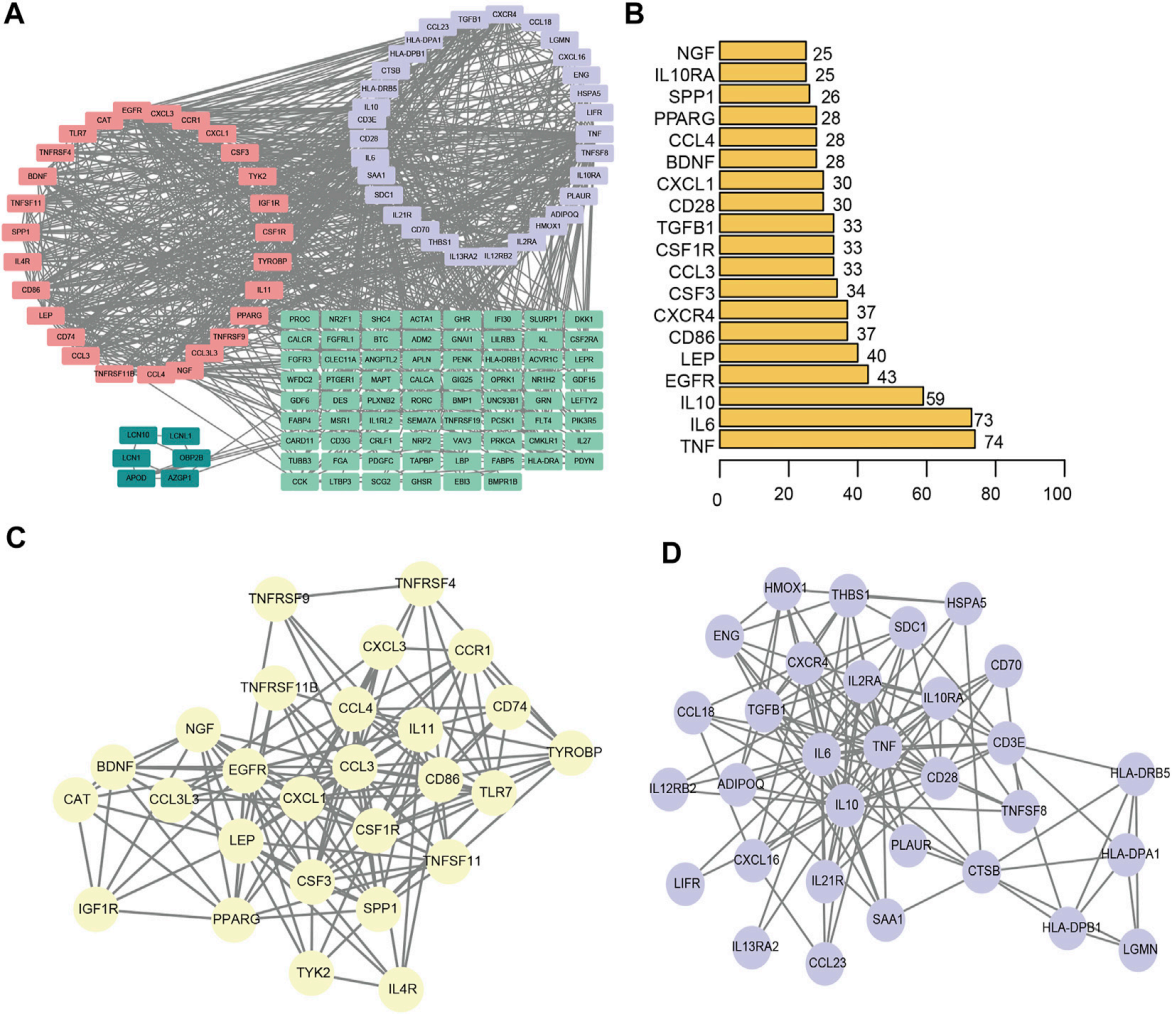
PPI network. **(A)** PPI network of DEGs associated with LFH immunity. Different colors indicate different modules. **(B)** The number of interactions between the top 20 significant nodes in the PPI network and other genes; vertical coordinates indicate the DEGs associated with LFH immunity, and the horizontal coordinates are the number of pairs of interactions between each of these DEGs and other genes. **(C)** MCODE identified key module 1 in the PPI network. **(D)** MCODE identified key module 2 in the PPI network.

We constructed a protein-protein interaction network of differentially expressed genes associated with the immunity of ligamentum flavum hypertrophy (Figure 7A). A total of 144 immune-related DEGs associated with LFH and 877 PPI pairs were found. The top five DEGs with most interactions with other LFH immune-related DEGs were tumor necrosis factor (*TNF*; 74 interactions), *IL6* (73 interactions), *IL10* (59 interactions), epidermal growth factor receptor (*EGFR;* 43 interactions), and leptin (*LEP*; 40 interactions) (Figure 7B). The MCODE plug-in used to cluster the PPI networks revealed two key modules (Figures 7C,D) and used the 27 genes in module 1 (score > 10) as the key DEGs associated with LFH immunity.

### Friends analysis

**FIGURE 8:**
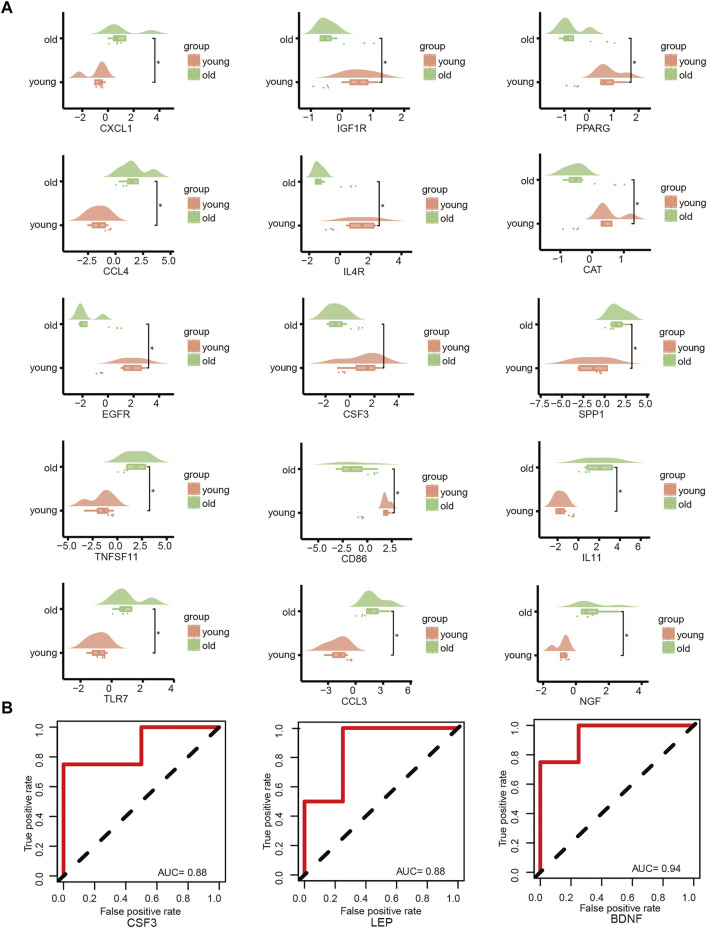
Key gene analysis. **(A)** Cloud and rain plots show the expression differences of LFH immune-related DEGs in the elderly and young groups; vertical coordinates indicate the grouping and horizontal coordinates the expression levels of the different DEGs. **(B)** ROC curves of the key genes *CSF3*, *LEP*, and *BDNF* in the GSE113212 dataset; only genes with AUC values above 0.7 are shown.

**FIGURE 9:**
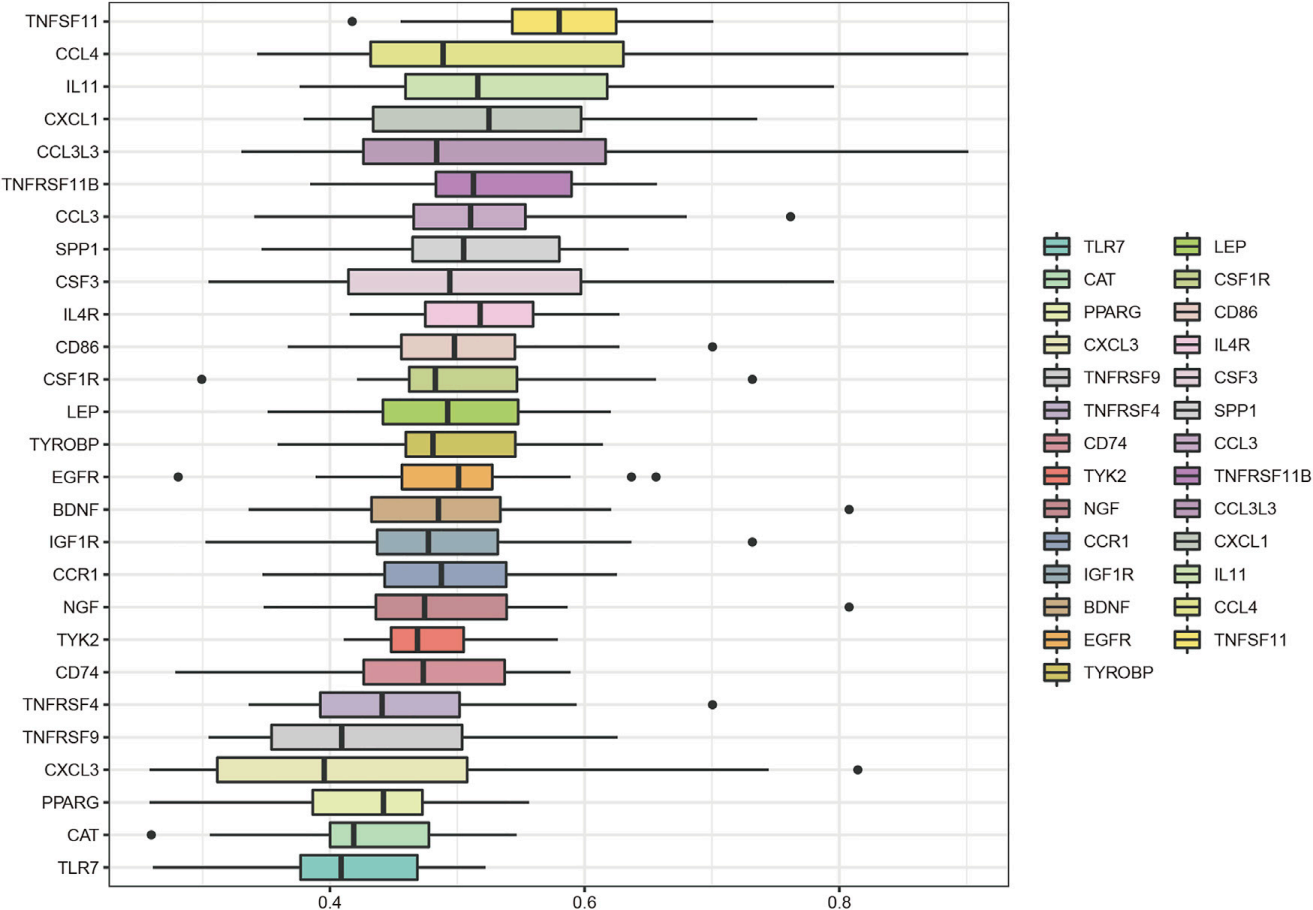
Friends analysis. The box plot shows the functional similarity of the DEGs associated with LFH immunity with other genes. *TNFSF11* showed the strongest correlation with other genes.

The key genes associated with LFH immunity were differentially expressed between the elderly LFH and young normal LF groups (Figure 8A). The ROC curves of brain-derived neurotrophic factor (*BDNF*), colony-stimulating factor 3 (*CSF3*), and *LEP* genes had AUC values greater than 0.8, indicating the potential of these three genes to distinguish between the LFH and normal LF groups (Figure 8B). The Friends analysis performed for key DEGs associated with LFH immunity showed that TNF superfamily member 11 (*TNFSF11*) had the strongest correlation with the other DEGs and may thus play a key role in LFH immunity (Figure 9).

### Immune infiltration correlation analysis

**FIGURE 10:**
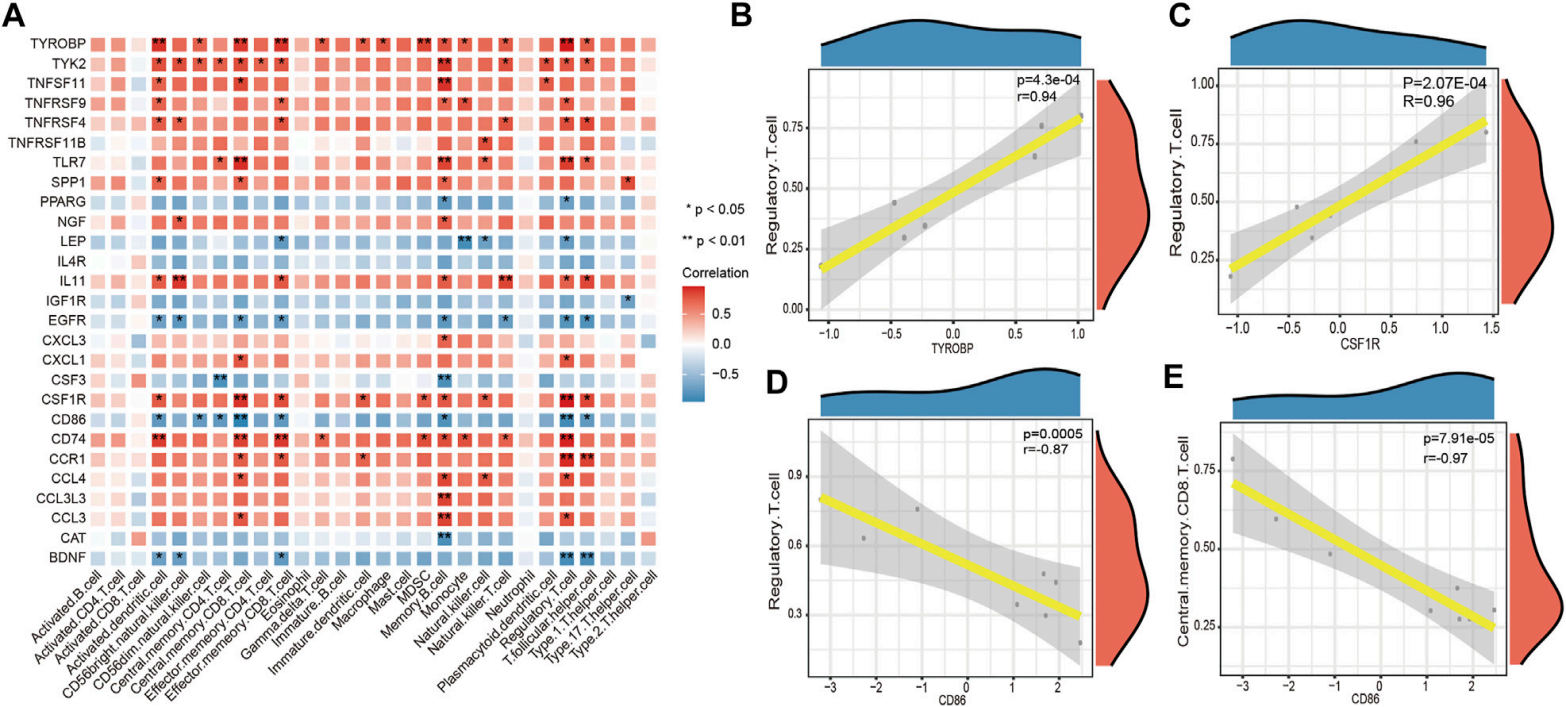
Immune cell infifiltration correlation analysis. **(A)** Heatmap showing the results of correlation analysis between the DEGs associated with LFH immunity and infifiltrated immune cells. The horizontal axis indicates the different immune cell types, and the vertical axis indicates the DEGs associated with LFH; the darker the color of the square, the stronger the correlation between the DEGs and the immune cell type; * indicates the difference is statistically signifificant (*p* < 0.05); **(B, C)** scatter plots of the top 2 positively correlated gene pairs with immune cells. **(D, E)** scatter plots of the top 2 negatively correlated gene pairs with immune cells. Horizontal coordinates indicate the DEG expression levels and vertical coordinates the ssGSEA enrichment scores of immune cell types.

The correlation between key DEGs associated with LFH immunity and immune cell infiltration revealed several significant correlations (Figure 10). For example, there were significant negative correlations between cluster of differentiation 86 (*CD86*) and central memory CD8 T cells and regulatory T cells, and significant positive correlations between *CSF1R* and tyrosine kinase-binding protein (*TYROBP*) genes and regulatory T cells.

### Quantitative reverse transcription-polymerase chain reaction and immunohistochemical assessments validation of bioinformatics results

**FIGURE 11:**
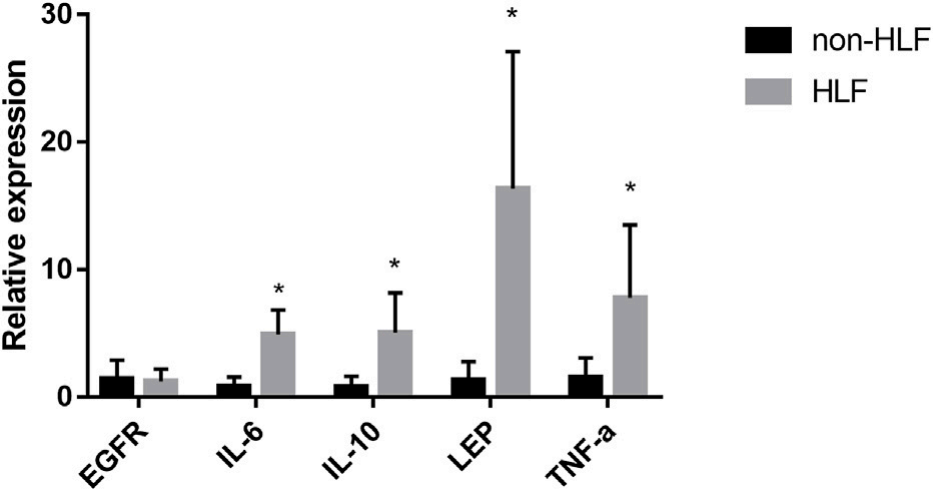
The mRNA relative expression levels of *EGFR, IL-6, IL-10, LEP*, and *TNF- α* in the LFH(*n* = 6) and normal LF groups (*n* = 6) were verified by qRT-PCR operated in 40 cycles, and the expression was calculated using the 2^−**∆∆**CT^ method. The expression of *IL-6, IL-10, LEP*, and *TNF-α* was significantly higher in the LFH group than in the normal LF group (*, *p* < 0.05) but there was no difference in *EGFR* expression.

**FIGURE 12:**
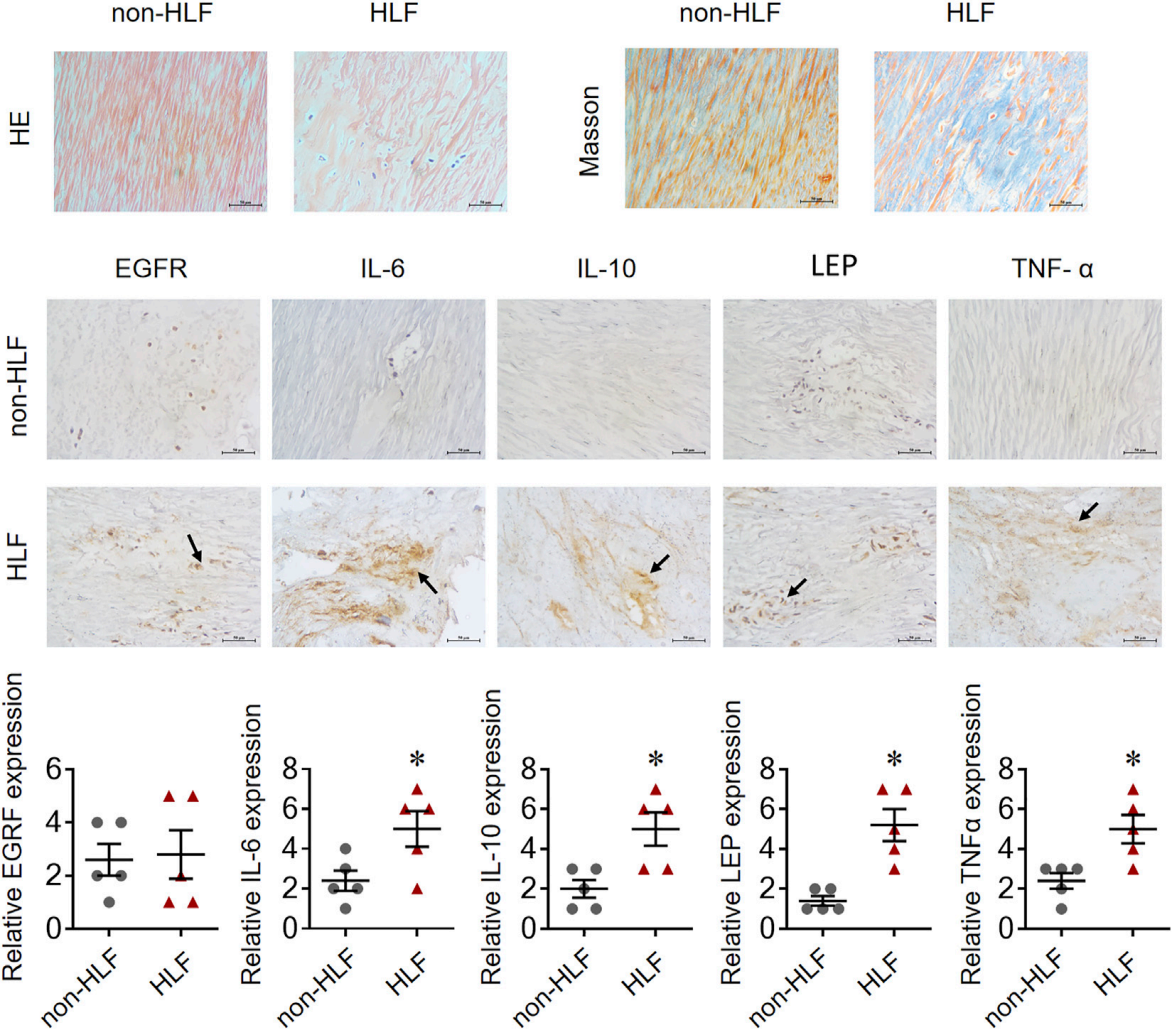
EGFR, IL-6, IL-10, LEP, and TNF-α expression in representative LF tissue from LFH (*n* = 5)and normal LF group (*n* = 5) verified by IHC.The expression of IL-6, IL-10, LEP, and TNF-α was significantly higher in the LFH group than in the normal LF group (*, *p* < 0.05, *n* = 5) but there was no difference in EGFR expression.

The mRNA levels of *IL-6, IL-10, LEP*, and *TNF-α* obtained using qRT-PCR were significantly higher in the LFH tissues than in the normal LF tissues, with LEP presenting the most notable differential expression. However, there was no significant difference in EGFR mRNA expression between the two groups (Figure 11). We also confirmed the above results in histopathology (Figure 12). Overall, these results corroborate the reliability of the bioinformatics results.

## Discussion

Ligamentum flavum hypertrophy is a major cause of lumbar spinal stenosis and the pathology includes fibrosis with increased elastic fiber rupture, cell count and collagen. However, there is no exact target or therapeutic mechanism for ligamentum flavum hypertrophy, so finding markers of ligamentum flavum hypertrophy is urgent. In the present study, the 1530 DEGs obtained from GSE113212 were subjected to pathway enrichment analysis; ECM-receptor interaction, cytokine-cytokine receptor interaction, and PI3K-Akt signaling pathway were among the most enriched biological pathways. The top five DEGs with the most gene interactions were TNF-α*, IL6, IL10, EGFR*, and *LEP,* and a significant correlation was found between key DEGs (or hub genes) associated with LFH immunity and several immune cell types. For instance, *CD86* showed a significant negative correlation with central memory CD8 T cells and regulatory T cells, while *CSR1R* and *TYROBP* genes showed significant positive correlations with regulatory T cells. qRT-PCR was used to measure the mRNA levels of IL-6, IL-10, LEP, and TNF-α in LFH tissues. IL-6, IL-10, LEP, and TNF-α mRNA levels were considerably greater than in normal LF tissues.

Numerous functions have been described for TNF, a ubiquitously expressed proinflammatory cytokine (Kroetsch et al., 2017) supporting the inflammatory response (Deng et al., 2018). Increased expression of TNF is associated with osteogenesis of the LF, which contributes to spinal stenosis (Lin et al., 2018). TNF-α stimulated IL-6 release by ligamentum flavum-derived cells is thought to be mediated by p38 MAPK (Sun et al., 2018). Preliminary studies have shown that the COX-2 inhibitor celecoxib reduces chronic mechanical pain induced by degenerative disc disease in rats (Lee et al., 2019). Reduced miR-221 expression may enhance LF hypertrophy by increasing TIMP-2 expression, which in turn raises the levels of type I and III collagen in the body. In addition, we found that miR-221 may be a new therapeutic target for LSS based on our findings (Xu et al., 2016). The expression IL-10, an anti-inflammatory cytokine, is enhanced during cytokine storms and may facilitate self-protection. Immunosuppression in sepsis is related to high levels of IL-10, which is directly linked to inflammation (Zhu et al., 2020). Intervertebral disc degeneration and stenosis are also associated with changes in IL-10 levels in the lumbar ligament (Martins et al., 2019).

Both GO and KEGG analysis revealed that DEGs associated with LFH were particularly enriched in cytokine activity-related and PI3K-Akt signaling pathways. By activating the PI3K/Akt pathway, the metalloproteinase ADAM10 has been shown to increase cell proliferation and hypertrophy in LF cells *in vitro* (Pan et al., 2021). Previous research demonstrated that inflammatory responses initiate the PI3K/Akt signaling pathway, (He et al., 2020) which is an important signaling pathway for cell survival (He et al., 2020). Knockdown of Forkhead box protein O1 decreased osteogenic development of human lumbar ligament cells (Hao et al., 2018). Components of the signaling pathway, which controls the organ size of animals via the regulation of cell proliferation and apoptosis, include growth differentiation factor 5 and the segment polarity protein disheveled homolog 2 that are implicated in the osteogenic differentiation of LF-derived stem cells and synovial fibroblasts, respectively (Wu et al., 2019). In LF cells, synoviocytes, and melanoma cell lines, elastin-derived peptides and/or VGVAPG peptides have been demonstrated to promote the synthesis and/or secretion of inflammatory markers such as IL-1 and IL-6 (Szychowski et al., 2020). The differentially expressed genes linked with ligamentum flavum hypertrophy in the current study were mostly enriched in immunological synapses, lysosomal compartments, immune receptor activity, integrin binding, and ECM receptor interactions, which is a new finding. However, because this is the first study to link ligamentum flavum hypertrophy-associated genes to ECM receptor interactions, the regulatory mechanisms must be investigated further.

Immune-related differentially expressed genes play an important role in ligamentum flavum hypertrophy, we turned to bioinformatics analysis to examine how the disease’s differentially expressed genes interact with one another. Ligamentum flavum hypertrophy may be explained by the explanation of TNF-α, IL6, IL10, and LEP, which we designated as prospective targets. Several essential signaling pathways and biological activities were regulated by the hub genes found throughout the screening process. In addition to its role in the immune system, the tumor necrosis factor (TNF) has been implicated in a variety of other biological processes. TNF-α (tumor necrosis factor α) is a cytokine that stimulates inflammation (Kroetsch et al., 2017). TNF α, a pro-inflammatory cytokine, can cause the ligamentum flavum to ossify (Lin et al., 2018; Sugano et al., 2019). In humans, leptin (LEP) encodes the peptide hormone leptin (homolog of the mouse obesity gene) (Min et al., 2019). Obesity and enhanced bone metabolism are linked to adipokines like leptin, and obese rats with elevated leptin receptor genes experience spinal ligament ossification, according to research (Oudkerk et al., 2019). IL-6 is a member of the cytokine IL-6 family (Dittmer et al., 2020). Fibroblastic ligament cells express high levels of IL-6, which is a pro-inflammatory cytokine (Bageghni et al., 2018).

We used the CIBERSORT algorithm to calculate the degree of infiltration of 22 immune cells in the aged ligamentum flavum hypertrophy group and the young ligamentum normal group. T cells CD4 memory activated and B cells naïve were significantly different, and T cells CD4 memory activated and B cells naïve. Macrophages. M1 and T.cells. CD8 cells showed a significant positive correlation. A mouse model of ligamentum flavum hypertrophy was used in one study to investigate disease-related variables. Ligamentum flavum hypertrophy was demonstrated 6 weeks following microinjury to the ligamentum flavum in mice, which induced macrophage infiltration and collagen synthesis (Saito et al., 2017b). Further hypertrophy may be perpetuated by macrophages, which have been discovered to be a key biological source of TGFin in patients with advanced LFH (Löhr et al., 2011). Collagen gene expression was considerably higher in macrophage-infiltrated laser microdissected tissue than in undamaged fibroblasts in LFH microdamaged cells, as shown by quantitative RT-PCR. LFH hypertrophy may be triggered by an increase in fibroblast collagen synthesis due to infiltration of macrophages, according to these researches (Saito et al., 2017a). The above findings are consistent with this paper; in this study, we found that T cells CD4 memory activated and B cells naïve are new immune infiltrating cells identified in this study, which are not currently reported in ligamentum flavum hypertrophy and are innovative.

Although we can only establish the clinical importance of our model based on existing data from real-world settings, it is worth mentioning that the present study assessed the mechanisms and biological pathways of LFH *in vitro*. Further research is therefore needed to fully examine the unique processes of LFH using a large multicenter sample to overcome the limitations of the present study, namely, the single data source and small sample size.

In summary, the DEGs and critical molecular pathways of LFH were identified using a comprehensive bioinformatics study of the GSE113212 LFH dataset and confirmed by the higher expression levels of *IL-6*, *IL-10*, *LEP*, and *TNF*-α in human LFH tissues *in vitro* using qRT-PCR and IHC. In this investigation, immune infiltrating cells that have not before been reported in ligamentum flavum hypertrophy—T cells CD4 memory activated and B cells nave—were discovered. Particularly abundant in cytokine activity-related and PI3K-Akt signaling pathways were DEGs linked to LFH. This novel information on the molecular mechanisms of LFH may aid in the development of novel therapeutic targets and approaches. However, the results obtained here need to be further confirmed using *in vitro* and animal studies and large sample size.

## Data Availability

The datasets analyzed in this study can be found in the GSE113212 data set of the Gene Expression Omnibus database.

## References

[B1] Amudong A. Muheremu A. Abudourexiti T. (2017). Hypertrophy of the ligamentum flavum and expression of transforming growth factor beta. J. Int. Med. Res. 45 (6), 2036–2041. 10.1177/0300060517711308 28635357 PMC5805210

[B2] Bageghni S. A. Hemmings K. E. Zava N. Denton C. P. Porter K. E. Ainscough J. F. X. (2018). Cardiac fibroblast-specific p38α MAP kinase promotes cardiac hypertrophy via a putative paracrine interleukin-6 signaling mechanism. Faseb J. 32 (9), 4941–4954. 10.1096/fj.201701455RR 29601781 PMC6629170

[B3] Barrett T. Troup D. B. Wilhite S. E. Ledoux P. Rudnev D. Evangelista C. (2007). NCBI GEO: Mining tens of millions of expression profiles--database and tools update. Nucleic Acids Res. 35, D760–D765. 10.1093/nar/gkl887 17099226 PMC1669752

[B4] Bhattacharya S. Andorf S. Gomes L. Dunn P. Schaefer H. Pontius J. (2014). ImmPort: Disseminating data to the public for the future of immunology. Immunol. Res. 58 (2-3), 234–239. 10.1007/s12026-014-8516-1 24791905

[B5] Chen B. Khodadoust M. S. Liu C. L. Newman A. M. Alizadeh A. A. (2018). Profiling tumor infiltrating immune cells with CIBERSORT. Methods Mol. Biol. 1711, 243–259. 10.1007/978-1-4939-7493-1_12 29344893 PMC5895181

[B6] Chen J. Liu Z. Wang H. Qian L. Li Z. Song Q. (2021). SIRT6 enhances telomerase activity to protect against DNA damage and senescence in hypertrophic ligamentum flavum cells from lumbar spinal stenosis patients. Aging (Albany NY) 13 (4), 6025–6040. 10.18632/aging.202536 33568575 PMC7950242

[B7] Cisneros J. A. Robertson M. J. Valhondo M. Jorgensen W. L. (2016). A fluorescence polarization assay for binding to macrophage migration inhibitory factor and crystal structures for complexes of two potent inhibitors. J. Am. Chem. Soc. 138 (27), 8630–8638. 10.1021/jacs.6b04910 27299179 PMC4945996

[B8] Davis S. Meltzer P. S. (2007). GEOquery: A bridge between the gene expression omnibus (GEO) and BioConductor. Bioinformatics 23 (14), 1846–1847. 10.1093/bioinformatics/btm254 17496320

[B9] Deng X. Zhang X. Tang B. Liu H. Shen Q. Liu Y. (2018). Design, synthesis, and evaluation of dihydrobenzo[cd]indole-6-sulfonamide as TNF-α inhibitors. Front. Chem. 6, 98. 10.3389/fchem.2018.00098 29670876 PMC5893771

[B10] Dittmer A. Lange T. Leyh B. Dittmer J. (2020). Protein- and growth-modulatory effects of carcinoma-associated fibroblasts on breast cancer cells: Role of interleukin-6. Int. J. Oncol. 56 (1), 258–272. 10.3892/ijo.2019.4918 31789400 PMC6910226

[B11] Hänzelmann S. Castelo R. Guinney J. (2013). GSVA: Gene set variation analysis for microarray and RNA-seq data. BMC Bioinforma. 14, 7. 10.1186/1471-2105-14-7 PMC3618321 23323831

[B12] Hao W. Liu H. Zhou L. Sun Y. Su H. Ni J. (2018). MiR-145 regulates osteogenic differentiation of human adipose-derived mesenchymal stem cells through targeting FoxO1. Exp. Biol. Med. 243 (4), 386–393. 10.1177/1535370217746611 PMC6022928 29249185

[B13] He P. Ma J. Liu Y. Deng H. Dong W. (2020). Hesperetin promotes cisplatin-induced apoptosis of gastric cancer *in vitro* and *in vivo* by upregulating PTEN expression. Front. Pharmacol. 11, 1326. 10.3389/fphar.2020.01326 32973533 PMC7482524

[B14] Kaufman H. H. Ommaya A. K. Dopman J. L. Roth J. A. (1971). Hypertrophy of the ligamentum flavum. Secondary cord syndrome in an acromegalic. Arch. Neurol. 25 (3), 256–259. 10.1001/archneur.1971.00490030082009 4951948

[B15] Kroetsch J. T. Levy A. S. Zhang H. Aschar-Sobbi R. Lidington D. Offermanns S. (2017). Constitutive smooth muscle tumour necrosis factor regulates microvascular myogenic responsiveness and systemic blood pressure. Nat. Commun. 8, 14805. 10.1038/ncomms14805 28378814 PMC5382284

[B16] Lee J. Y. Choi H. Y. Park C. S. Jang C. Lee K. T. Lee J. Y. (2019). Inhibition of COX-2 alleviates lumbar spinal stenosis-induced chronic mechanical allodynia in rats. Int. Immunopharmacol. 75, 105738. 10.1016/j.intimp.2019.105738 31306980

[B17] Liberzon A. Birger C. Thorvaldsdottir H. Ghandi M. Mesirov J. P. Tamayo P. (2015). The Molecular Signatures Database (MSigDB) hallmark gene set collection. Cell Syst. 1 (6), 417–425. 10.1016/j.cels.2015.12.004 26771021 PMC4707969

[B18] Lin B. Srikanth P. Castle A. C. Nigwekar S. Malhotra R. Galloway J. L. (2018). Modulating cell fate as a therapeutic strategy. Cell Stem Cell 23 (3), 329–341. 10.1016/j.stem.2018.05.009 29910150 PMC6128730

[B19] Löhr M. Hampl J. A. Lee J. Y. Ernestus R. I. Deckert M. Stenzel W. (2011). Hypertrophy of the lumbar ligamentum flavum is associated with inflammation-related TGF-β expression. Acta Neurochir. 153 (1), 134–141. 10.1007/s00701-010-0839-7 20960015

[B20] Lu C. Liu Z. Zhang H. Duan Y. Cao Y. (2019). Mechanism of p38 mitogen activated protein kinase signaling pathway on promoting the hypertrophy of human lumbar ligamentum flavum via transforming growth factor β (1)/connective tissue growth factor. Zhongguo Xiu Fu Chong Jian Wai Ke Za Zhi 33 (6), 730–735. 10.7507/1002-1892.201811140 31198002 PMC8355761

[B21] Ma C. Qi X. Wei Y. F. Li Z. Zhang H. L. Li H. (2023). Amelioration of ligamentum flavum hypertrophy using umbilical cord mesenchymal stromal cell-derived extracellular vesicles. Bioact. Mat. 19, 139–154. 10.1016/j.bioactmat.2022.03.042 PMC9014323 35475028

[B22] Martins D. E. Wajchenberg M. Veridiano J. M. Theodoro T. R. Toledo O. M. S. Pinhal M. A. S. (2019). Molecular alterations of human lumbar yellow ligament related to the process of intervertebral disk degeneration and stenosis. Eur. Spine J. 28 (6), 1413–1422. 10.1007/s00586-019-05994-3 31069526

[B23] Min D. Y. Jung E. Kim J. Lee Y. H. Shin S. Y. (2019). Leptin stimulates IGF-1 transcription by activating AP-1 in human breast cancer cells. BMB Rep. 52 (6), 385–390. 10.5483/bmbrep.2019.52.6.189 30293548 PMC6605521

[B24] Oudkerk S. F. Mohamed Hoesein F. A. A. PThM Mali W. Oner F. C. Verlaan J. J. de Jong P. A. (2019). Subjects with diffuse idiopathic skeletal hyperostosis have an increased burden of coronary artery disease: An evaluation in the COPDGene cohort. Atherosclerosis 287, 24–29. 10.1016/j.atherosclerosis.2019.05.030 31181416 PMC8041152

[B25] Pan B. Huo T. Cao M. Jing L. Luo X. Qu Z. (2021). ADAM10 promotes the proliferation of ligamentum flavum cells by activating the PI3K/AKT pathway. Int. J. Mol. Med. 47 (2), 688–698. 10.3892/ijmm.2020.4809 33416124 PMC7797459

[B26] Park J. B. Lee J. K. Park S. J. Riew K. D. (2005). Hypertrophy of ligamentum flavum in lumbar spinal stenosis associated with increased proteinase inhibitor concentration. J. Bone Jt. Surg. Am. 87 (12), 2750–2757. 10.2106/JBJS.E.00251 16322626

[B27] Ritchie M. E. Phipson B. Wu D. Hu Y. Law C. W. Shi W. (2015). Limma powers differential expression analyses for RNA-sequencing and microarray studies. Nucleic Acids Res. 43 (7), e47. 10.1093/nar/gkv007 25605792 PMC4402510

[B28] Ruberg F. L. Grogan M. Hanna M. Kelly J. W. Maurer M. S. (2019). Transthyretin amyloid cardiomyopathy: JACC state-of-the-art review. J. Am. Coll. Cardiol. 73 (22), 2872–2891. 10.1016/j.jacc.2019.04.003 31171094 PMC6724183

[B29] Saito T. Hara M. Kumamaru H. Kobayakawa K. Yokota K. Kijima K. (2017a). Macrophage infiltration is a causative factor for ligamentum flavum hypertrophy through the activation of collagen production in fibroblasts. Am. J. Pathol. 187 (12), 2831–2840. 10.1016/j.ajpath.2017.08.020 28935572

[B30] Saito T. Yokota K. Kobayakawa K. Hara M. Kubota K. Harimaya K. (2017b). Experimental mouse model of lumbar ligamentum flavum hypertrophy. PLoS One 12 (1), e0169717. 10.1371/journal.pone.0169717 28060908 PMC5217959

[B31] Sing T. Sander O. Beerenwinkel N. Lengauer T. (2005). ROCR: Visualizing classifier performance in R. Bioinformatics 21 (20), 3940–3941. 10.1093/bioinformatics/bti623 16096348

[B32] Subramanian A. Tamayo P. Mootha V. K. Mukherjee S. Ebert B. L. Gillette M. A. (2005). Gene set enrichment analysis: A knowledge-based approach for interpreting genome-wide expression profiles. Proc. Natl. Acad. Sci. U. S. A. 102 (43), 15545–15550. 10.1073/pnas.0506580102 16199517 PMC1239896

[B33] Sugano T. Seike M. Funasaka Y. Yoshida M. Takayama R. Okamura K. (2019). Intralymphatic histiocytosis in a patient with lung adenocarcinoma treated with pembrolizumab: A case report. J. Immunother. Cancer 7 (1), 59. 10.1186/s40425-019-0534-z 30813943 PMC6391791

[B34] Sun C. Wang Z. Tian J. W. Wang Y. H. (2018). Leptin-induced inflammation by activating IL-6 expression contributes to the fibrosis and hypertrophy of ligamentum flavum in lumbar spinal canal stenosis. Biosci. Rep. 38 (2), BSR20171214. 10.1042/BSR20171214 29436483 PMC5874260

[B35] Szklarczyk D. Gable A. L. Lyon D. Junge A. Wyder S. Huerta-Cepas J. (2019). STRING v11: Protein-protein association networks with increased coverage, supporting functional discovery in genome-wide experimental datasets. Nucleic Acids Res. 47 (D1), D607–D613. 10.1093/nar/gky1131 30476243 PMC6323986

[B36] Szychowski K. A. Skora B. Tobiasz J. Gminski J. (2020). Elastin-derived peptide VGVAPG decreases differentiation of mouse embryo fibroblast (3T3-L1) cells into adipocytes. Adipocyte 9 (1), 234–245. 10.1080/21623945.2020.1770525 32463311 PMC7469433

[B37] Wang C. Yin X. Zhang L. Xue X. Xiang Y. Jin H. (2020). Posterolateral fusion combined with posterior decompression shows superiority in the treatment of severe lumbar spinal stenosis without lumbar disc protrusion or prolapse: A retrospective cohort study. J. Orthop. Surg. Res. 15 (1), 26. 10.1186/s13018-020-1552-8 31969171 PMC6977327

[B38] Wang L. Chang M. Tian Y. Yan J. Xu W. Yuan S. (2021). The role of Smad2 in transforming growth factor β(1)-induced hypertrophy of ligamentum flavum. World Neurosurg. 151, e128–e136. 10.1016/j.wneu.2021.03.147 33831616

[B39] Wu Y. Ou Y. Liao C. Liang S. Wang Y. (2019). High-throughput sequencing analysis of the expression profile of microRNAs and target genes in mechanical force-induced osteoblastic/cementoblastic differentiation of human periodontal ligament cells. Am. J. Transl. Res. 11 (6), 3398–3411. 31312353 PMC6614645

[B40] Xu Y. Q. Zhang Z. h. Zheng Y. f. Feng S. q. (2016). MicroRNA-221 regulates hypertrophy of ligamentum flavum in lumbar spinal stenosis by targeting TIMP-2. Spine (Phila Pa 1976) 41 (4), 275–282. 10.1097/BRS.0000000000001226 26571175

[B41] Yu G. (2020). Gene ontology semantic similarity analysis using GOSemSim. Methods Mol. Biol. 2117, 207–215. 10.1007/978-1-0716-0301-7_11 31960380

[B42] Yu G. Wang L. G. Han Y. He Q. Y. (2012). clusterProfiler: an R package for comparing biological themes among gene clusters. Omics 16 (5), 284–287. 10.1089/omi.2011.0118 22455463 PMC3339379

[B43] Zhang B. Wu Q. Li B. Wang D. Wang L. Zhou Y. L. (2020). m(6 A regulator-mediated methylation modification patterns and tumor microenvironment infiltration characterization in gastric cancer. Mol. Cancer 19 (1), 53. 10.1186/s12943-020-01170-0 32164750 PMC7066851

[B44] Zheng Z. Ao X. Li P. Lian Z. Jiang T. Zhang Z. (2020). CRLF1 is a key regulator in the ligamentum flavum hypertrophy. Front. Cell Dev. Biol. 8, 858. 10.3389/fcell.2020.00858 33072735 PMC7533558

[B45] Zhu Z. Cai T. Fan L. Lou K. Hua X. Huang Z. (2020). Clinical value of immune-inflammatory parameters to assess the severity of coronavirus disease 2019. Int. J. Infect. Dis. 95, 332–339. 10.1016/j.ijid.2020.04.041 32334118 PMC7195003

